# A novel indicator in evaluating endoscopic orbital decompression for thyroid-associated orbitopathy

**DOI:** 10.3389/fendo.2025.1527376

**Published:** 2025-04-08

**Authors:** Jieliang Shi, Zhaoqi Pan, Yunhai Tu, Zhirong Liu, Siyu Dong, Yuwan Gao, Wentao Li, Jian Yang, Wencan Wu

**Affiliations:** ^1^ Eye Hospital of Wenzhou Medical University, Wenzhou, Zhejiang, China; ^2^ School of Computer Science and Technology, Beijing Institute of Technology, Beijing, China; ^3^ School of Optics and Photonics, Beijing Institute of Technology, Beijing, China

**Keywords:** thyroid-associated orbitopathy, effective orbital volume, artificial intelligence technology, orbital decompression, lateral wall decompression

## Abstract

**Objective:**

To introduce the increase rate of effective orbital volume (IREOV) as a novel indicator for evaluating outcomes of endoscopic orbital decompression in thyroid-associated orbitopathy (TAO).

**Methods:**

In this retrospective study, 69 subjects (124 orbits) underwent either medial wall decompression (MWD) or lateral wall decompression (LWD) combined with fat decompression for TAO. Artificial intelligence was used to segment the orbit and calculate IREOV to compare MWD and LWD effectiveness. The impact of postoperative extraocular muscle expansion on IREOV was also assessed, with patients categorized into muscle hypertrophy and fat hyperplasia groups.

**Results:**

Using Artificial Intelligence OrbitNet system, the average IREOV was 0.14 ± 0.08. Postoperative IREOV was significantly higher for MWD (0.17 ± 0.07) than for LWD (0.05 ± 0.05) (P<0.001). Medial rectus muscle expansion had the greatest impact on IREOV after MWD, while lateral rectus muscle expansion affected IREOV after LWD. Most importantly, TAO patients with muscle hypertrophy exhibited higher IREOV after MWD compared to those with fat hypertrophy (P<0.001). Proptosis reduction was 3.20 ± 1.37mm for MWD and 3.02 ± 0.68mm for LWD, with no significant difference (P>0.05).

**Conclusion:**

Accurate IREOV calculation through artificial intelligence is crucial for evaluating the efficacy of orbital decompression surgery. For TAO patients presenting with fat hyperplasia, LWD should be prioritized to minimize the risk of postoperative extraocular muscle expansion. In cases where MWD is performed on fat hyperplasia patients, rigorous postoperative surveillance for extraocular muscle expansion is essential.

## Introduction

Thyroid associated orbitopathy (TAO), characterized by swelling of the extraocular muscles and orbital fat, represents the prevailing orbital disease in adults (1). Orbital decompression, including medial wall decompression (MWD) and lateral wall decompression (LWD) combined with fat decompression, is a primary treatment option for TAO (2, 3). The main purpose of orbital decompression surgery is to increase orbital volume and reduce tissue congestion to achieve eyeball retraction. In clinical practice, surgical outcomes are typically evaluated by the degree of exophthalmos, extraocular muscle volume, fat volume, orbital volume, and other relevant parameters. These parameters are closely related to clinical manifestations, facilitating the effective management and evaluation of TAO patients.

Previously, ocular parameters such as Hertel exophthalmometry were measured manually. However, significant individual variations and low repeatability resulted in substantial measurement errors. Subsequently, computed tomography (CT) and magnetic resonance imaging (MRI) have proven effective in accurately assessing the extent of extraocular muscle and fat volume augmentation in TAO patients, as well as quantifying morphological alterations in the orbital region. Numerous previous studies have used CT, MRI and associated software to quantitatively evaluate orbit parameters. However, majority of these parameters were measured manually or adjusted using semi-automatic technology (4–10). The advent of artificial intelligence technology has enabled the automatic segmentation of orbital structures, calculation of orbital volume, and estimation of eyeball retraction, thereby reducing potential errors associated with manual measurements (11, 12).

Notably, in most patients undergoing orbital decompression, postoperative expansion of extraocular muscles is commonly observed, though the underlying mechanisms remain unclear (13–15). This muscle expansion occupies significant orbital space, hindering effective orbital volume expansion and affecting surgical outcomes (9, 16). Additionally, while orbital decompression surgery involves the removal of a certain volume of orbital fat, subsequent expansion of residual orbital fat tissue and orbital veins often occurs, making accurate measurement of these tissue volume changes challenging (17). Furthermore, studies have shown that orbital volume can vary significantly across racial, gender, and age groups, as identified through orbital CT analysis (18). Therefore, relying solely on increases in orbital volume to measure the effectiveness of orbital decompression is insufficient, as it does not account for postoperative extraocular muscles expansion and individual differences in orbital size. A more precise indicator for assessing surgical outcomes is greatly needed.

Thus, we introduced the concept of the increase rate of effective orbital volume (IREOV). IREOV is calculated as (postoperative orbital volume - postoperative organ volume) - (preoperative orbital volume - preoperative organ volume)/preoperative orbital volume. IREOV represents the expansion of orbital space achieved through bone removal, adjusted for the increased volume of extraocular muscles and the intrinsic size of the orbit. We believe that IREOV accurately reflects the actual impact of extraocular muscle expansion on orbital volume increase following decompression surgery. In this study, a comprehensive review and analysis of 94 cases of MWD and 31 cases of LWD performed at our center were conducted. By implementing artificial intelligence technology for organ segmentation, orbital related parameters were measured, and pertinent factors influencing the IREOV were analyzed, guiding the selection of appropriate surgical methods.

## Patients & methods

### Patients

In this observational retrospective study, TAO cases undergoing orbital wall decompression combined with fat decompression at the Eye Hospital of Wenzhou Medical University from March 2019 to August 2021 were reviewed. A total of 69 patients (124 orbits) were enrolled, of which 94 underwent MWD combined with fat decompression and 30 underwent LWD combined with fat decompression. All patients maintained a euthyroid state and were in the inactive stage of TAO at the time of surgery. Preoperative and postoperative orbital CT images and medical records, including complications and follow-ups, were reviewed. Clinical diagnosis was based on symptoms, signs, orbital CT images, and thyroid dysfunction history (19). Surgical indications included patients with stable TAO, defined as a Clinical Activity Score (CAS) of less than 3 for at least three months, and aesthetically significant exophthalmos with moderate protrusion (3-7 mm). Patients with dysthyroid optic neuropathy, severe exposure keratopathy, a history of major eye diseases, prior orbital surgery, trauma, radiotherapy, or unstable thyroid function were excluded from the study (20). Preoperative and postoperative orbital CT and complications during follow-up were evaluated.

The study adhered to the Declaration of Helsinki and was approved by the Ethics Committee of the Eye Hospital of Wenzhou Medical University. Written consent was obtained from all patients or their legal guardians.

### Surgical procedure

All procedures were completed by two experienced surgeons, Wencan Wu (Director of Ophthalmology) and Yunhai Tu (Director of Ophthalmology).Endoscopic medial orbital wall decompression combined with fat decompression (21) or endoscopic transconjunctival deep lateral wall decompression combined with fat decompression was performed (3) under general anesthesia with the patient supine.

### Postoperative management and follow-up

Intravenous methylprednisolone 500 mg/day was administered according to the specific conditions of each individual patient and intravenous broad‐spectrum antibiotics were given daily for two days post-surgery. Patients were advised to avoid vigorous activity, coughing, or sneezing for two weeks post-surgery. Exophthalmometry, visual acuity, visual field and eye movement were recorded during follow-up. Orbital CT was reexamined three months after surgery. Surgical complications were identified through reports and clinical records.

### IREOV calculation

**Figure 1:**
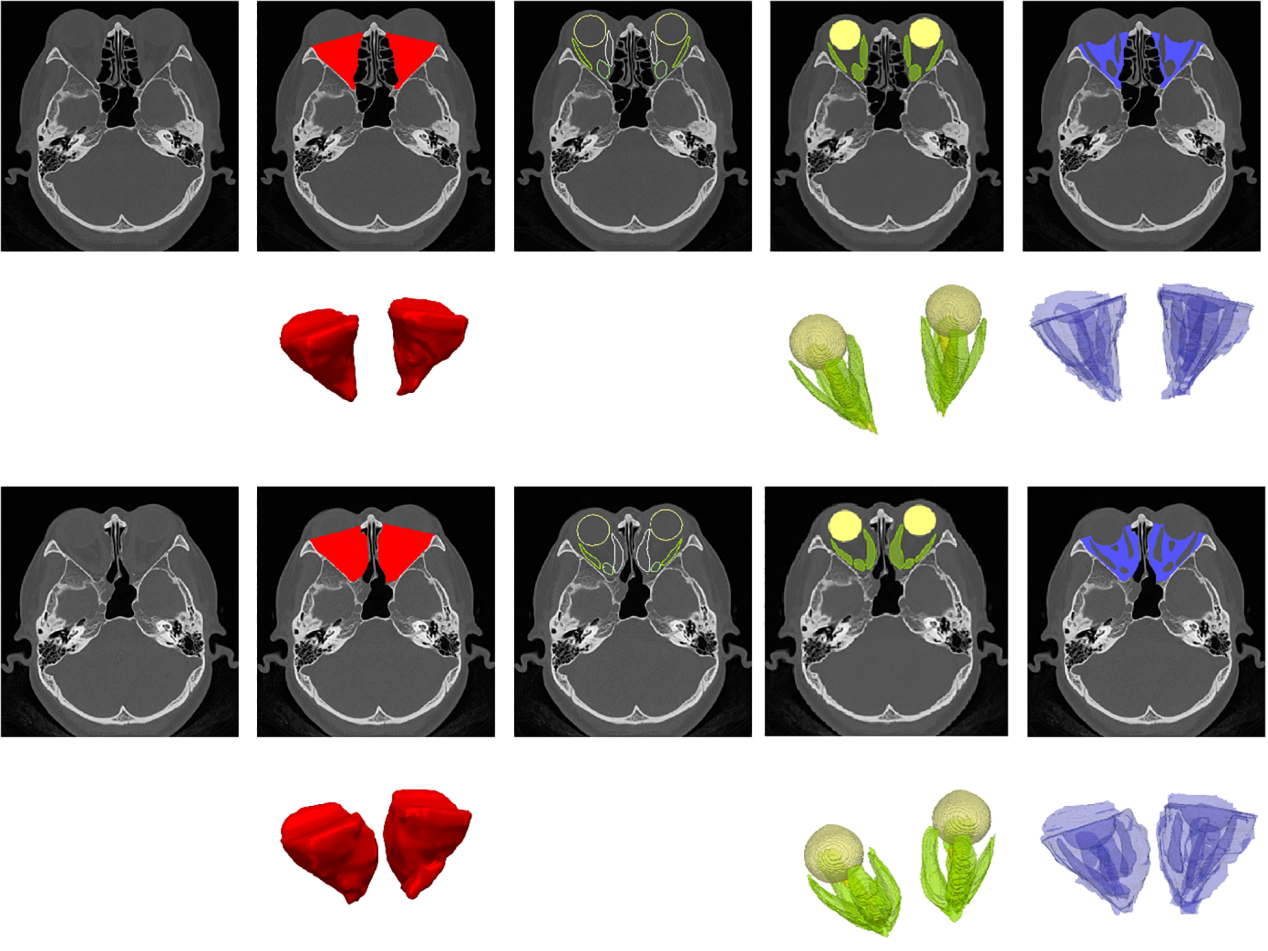
Illustration of orbit/organ segmentation by OrbitNet. This figure demonstrates the automatic segmentation of orbital organs using OrbitNet. The top row shows preoperative axial CT images, while the second row presents the segmented regions, color-coded as follows: red for orbital volume, green for the orbital organ volume, including the four rectus muscles, and blue for the volume after subtraction. The third and fourth rows display the corresponding postoperative axial CT images, with the segmented regions marked in the same color scheme.

**Figure 2:**
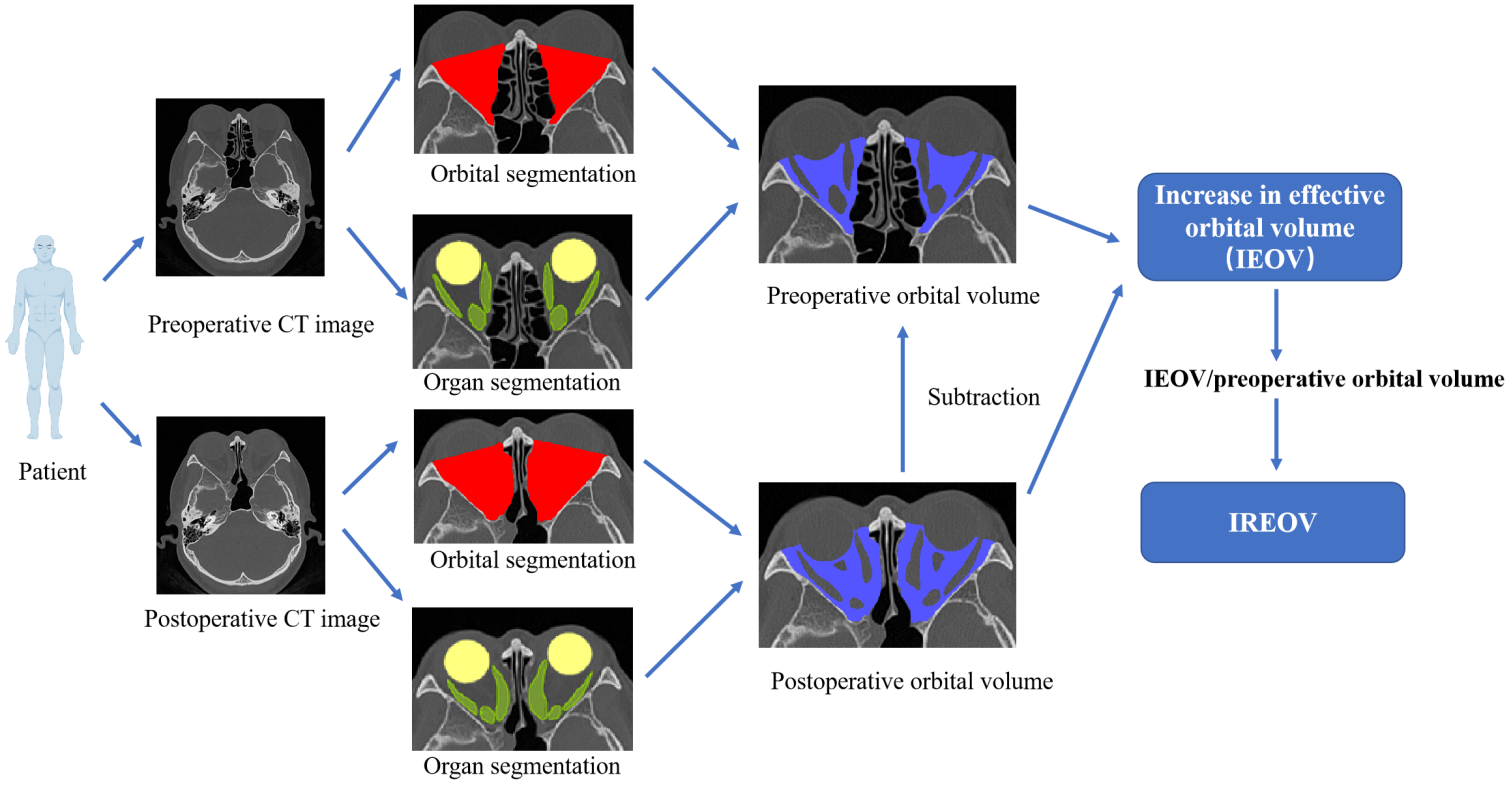
Schematic diagram of IREOV calculation through OrbitNet. The organ volume calculation includes the four rectus muscles.

An end-to-end automated deep learning model, OrbitNet, was previously developed by our team for accurate segmentation of the eyeball, optic nerve and rectus muscle (11, 12). The model includes a novel FocusTrans encoder that fuses global information to enhance feature extraction around target organs. Meanwhile, the model reduces segmentation instability caused by changes in the rectus muscle and optic nerve edges by using a hybrid loss function of structural similarity loss, achieving fully automatic accurate segmentation (**Figure 1**). The superior rectus muscle and levator palpebrae muscles were measured together due to difficulty separating them in images. After segmentation, the volume of each organ was calculated for statistical analysis (**Figure 2**).

### Exophthalmos measurement

A fully automatic 3D method based on CT images was used to measure eye proptosis distance. Traditional 2D CT methods acquire key points in the same slice, but most key points are not on the same CT slice. Our method based on three-dimensional CT selects optimal key points on different layers. Thus, it is more accurate than traditional 2D methods. The process is as follows:

1. The eyeball and surrounding orbital area are segmented based on CT images.
2. An automatic 3D segmentation algorithm is performed using 3D CT images to identify the most concave point on the upper and outer sides of the cheekbone as the base point.
3. The cross-sectional CT slice with the largest area of the eyeball is selected to determine the corneal apex.
4. The vertical distance between the corneal apex and the line connecting the concave points on the outer sides of the left and right cheekbones is calculated based on 3D segmentation. The change in this distance before and after surgery is defined as the exophthalmos distance.

### Statistical analysis

The paired sample t-test was used for normally distributed data sets, and the Wilcoxon test was used for nonparametric data using SPSS 22.0. A p-value of <0.05 indicated statistical significance, expressed as *p < 0.05, **p < 0.01, ***p < 0.001, and ****p < 0.0001.

## Results

A total of 69 patients (124 orbits) were enrolled in this study, with 38 (65 orbits) being female (**Table 1**). The average age of the patients was 50.81 ± 12.82 years (range, 22–75 years). The mean follow-up duration was 39.30 ± 8.01 months (range, 24–54 months). All patients exhibited varying degrees of exophthalmos.

**Table 1:** Demographic characteristics of the patients enrolled in this study.

Characteristics	N=69
Age (years)	50.81 ± 12.82
Sex (n)	
Female	38 (55.07%)
Male	31 (44.93%)
Follow-up duration (months)	39.30 ± 8.01
Medical background	
Smoking history (n)	5 (7.25%)
Diabetes history (n)	7 (10.14%)
Hypertension history (n)	14 (20.29%)
^131^I treatment history (n)	8 (11.59%)
History of glucocorticoid therapy (n)	18 (26.09%)
Thyroid	
Hyperthyroidism (%)	63 (91.30%)
Hypothyroidism (%)	0 (0.00%)
Normal (%)	6 (8.70%)
Duration of Thyroid dysfunction (months)	44.79 (0-240)
Duration of TAO (months)	12.26 (3-240)

**Figure 3:**
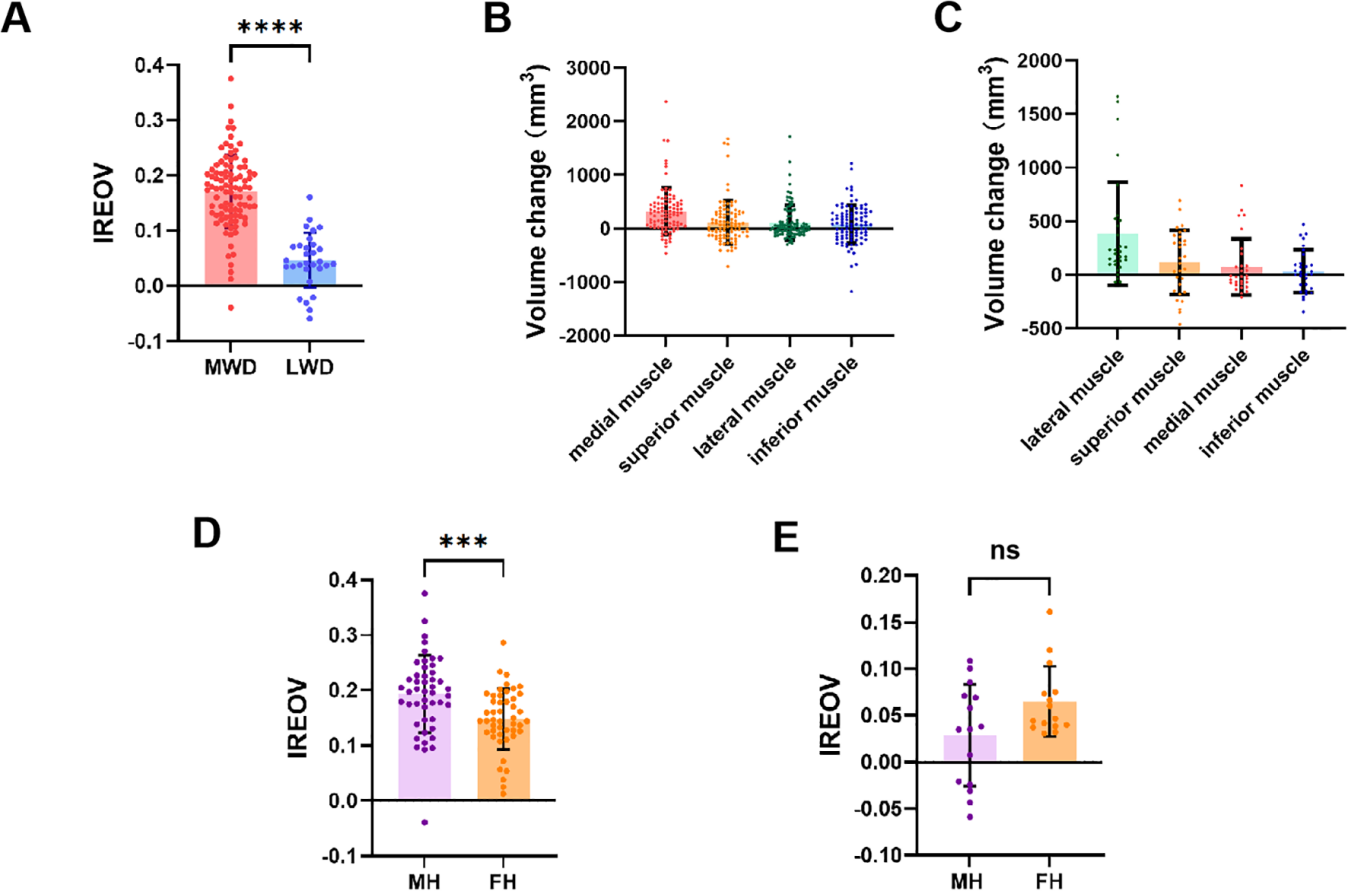
Relevant ophthalmological parameters of TAO patients. **(A)** Comparison of IREOV between MWD and LWD group. **(B)** The volume change of extraocular rectus muscles after MWD. **(C)** The volume change of extraocular rectus muscles after LWD. **(D)** Comparison of IREOV between muscle hypertrophy (MH) group and fat hyperplasia (FH) group in patients underwent MWD. **(E)** Comparison of IREOV between MH group and FH group in patients underwent LWD. ***p < 0.001, ****p < 0.0001.

The OrbitNet system was used to segment organs and calculate orbital parameters. The average IREOV was 0.14 ± 0.08 (range, -0.06–0.30). The IREOV measurements after MWD and LWD were 0.17 ± 0.07 (range, -0.04–0.30) and 0.05 ± 0.05 (range, -0.06–0.16), respectively. A statistically significant difference in IREOV between MWD and LWD was found (P<0.0001, **Figure 3A**), indicating that MWD was more effective in increasing orbital volume.

The impact of postoperative extraocular muscle expansion on IREOV was further studied, revealing that medial rectus muscle expansion following decompression had the most significant effect on IREOV after MWD (**Figure 3B**). In contrast, after LWD, the most notable effect on IREOV was from expansion of the lateral rectus muscle (**Figure 3C**). For further analysis, patients were divided into two groups: muscle hypertrophy and fat hyperplasia, based on orbital measurements and orbital CT findings. Patients with muscle hypertrophy exhibited a higher IREOV following MWD compared to those in the fat hyperplasia group (P<0.001, **Figure 3D**). However, for patients who underwent LWD, no significant difference in IREOV was observed between the two groups (P>0.05, **Figure 3E**).

Exophthalmometry was performed using the OrbitNet system based on HRCT by a single reviewer. An average postoperative reduction of 3.18 ± 1.32mm in proptosis was observed. For patients who underwent MWD, the reduction in proptosis was 3.20 ± 1.37mm, while for those who underwent LWD, the reduction was 3.02 ± 0.68mm. MWD was more effective than LWD in reducing proptosis, but the difference was not statistically significant (p>0.05).

Three patients developed new-onset diplopia after MWD and recovered through strabismus correction. No patients experienced intraoperative cerebrospinal fluid leakage, postoperative hemorrhage, infection, oculomotor or optic nerve injury, or globe dystopia during follow-up.

## Discussion

Orbital wall decompression and orbital fat decompression are the most commonly used surgical techniques for treating TAO (22). Orbital wall decompression increases the volume of the bony orbital cavity by expanding its enclosed space, while orbital fat decompression reduces the intraorbital content by removing fat, primarily from the intramuscular region. Together, these procedures alleviate crowding of orbital tissues caused by TAO, leading to the retraction of the eyeball. However, in several cases, orbital volume did not significantly increase, and eyeball retraction was unsatisfactory despite combined surgical intervention. A notable factor could be the substantial expansion of the extraocular muscles, occupying orbital space (14, 16). Additionally, orbital volume varies with race, age, gender, and orbital morphology (18, 23). Considering the thickening of extraocular muscles and individual differences in orbital size into account, we proposed the concept of IREOV to more accurately describe the increase in orbital volume from orbital decompression surgery.

Accurate segmentation of the eyeball, rectus muscle, and optic nerve is essential for precise calculation of IREOV. Previous studies measured orbital structures using manual segmentation, which had relatively large errors (24). Radiologists often spend several hours manually delineating organs. This process is time-consuming and requires a high level of expertise. Therefore, an end-to-end automated deep learning model, OrbitNet, was used to provide a more accurate quantitative analysis for recognizing and extracting organs. The model has been proven to surpass previous models in postoperative orbital segmentation (12). This approach saves time and reduces subjective errors, providing more objective and credible results. With OrbitNet, IREOV is easily calculated for further analysis.

The most common orbital wall decompression surgeries are medial and lateral orbital wall decompression (16). Different surgical techniques may have varying effects on muscles. In this study, we found that the IREOV of MWD was significantly higher than that of LWD. This discrepancy can be attributed to the different considerations involved in each procedure. MWD involves the removal of the medial orbital wall, allowing orbital tissue to herniate into the ethmoid sinus cavity, providing ample space. Conversely, LWD involves the removal of the greater wing of the sphenoid bone, resulting in less space. Therefore, although expansion of the extraocular muscles after MWD occupies more orbital volume, MWD is more effective in increasing orbital volume compared to LWD.

The study found that medial rectus muscles thickening following MWD had the most discernible effect on IREOV. The primary objective of modified medial wall decompression is to enlarge the medial space encompassing the medial rectus muscle (21). This procedure predominantly affects the medial rectus muscle, consistent with our findings. Additionally, the study found that expansion of the lateral rectus muscle after LWD had the most notable impact on IREOV. Transconjuctival deep LWD primarily creates additional space in the lateral orbital region, predominantly impacting the lateral rectus (3), aligning with our findings. Although there are various perspectives on the causes of extraocular muscle expansion following orbital decompression, it is possible that the surgery itself triggers a localized immune response within the orbit, leading to postoperative extraocular muscle expansion (15, 25).

In clinical practice, patients with TAO are commonly categorized into muscle hypertrophy type and fat hyperplasia type based on the predominant manifestation (18). The present study used orbital measurements and orbital CT findings to classify patients into two groups, referring to previous studies and clinical experience (26, 27). It was found that patients with muscular hyperplasia exhibited a higher IREOV following MWD, indicating that less muscle volume increase after surgery. This phenomenon could be attributed to muscle fibrosis in patients with muscular hyperplasia, limiting further muscle expansion despite enlarged space (28). Conversely, patients with fat hyperplasia are more likely to experience muscle expansion after surgery. Therefore, when opting for MWD in patients with fat hyperplasia, it is crucial to explain the potential risk of worsening postoperative muscle lesions before the procedure. Furthermore, no statistically significant difference in postoperative increase in orbital volume was observed between patients with muscle hypertrophy and those with fat hyperplasia following LWD. This may be due to the relatively limited expansion of orbital volume after LWD and the preservation of a thin layer of cortical bone in the lateral orbital wall, restricting further muscle hypertrophy.

The occurrence of a negative IREOV value requires careful consideration, as it suggests a decrease in effective orbital volume following decompression surgery. This could be due to various factors, including excessive postoperative expansion of extraocular muscles that occupy orbital space, limiting the intended increase in orbital volume. A negative IREOV may also reflect an insufficient or improper decompression procedure, where the volume increase is outweighed by muscle hypertrophy or residual orbital fat expansion. While the overall trend observed in this study was a positive IREOV after both MWD and LWD, the presence of negative values highlights the need for more precise patient selection, especially for those at risk of significant muscle expansion. Further research is required to better understand the mechanisms leading to negative IREOV and how these can be mitigated to ensure more predictable surgical outcomes in TAO patients.

Proptosis reduction is one of the most important purposes of surgery. A study by Li et al. indicated a significant correlation between changes in proptosis and intraconal volume in pure orbital fat decompression, but it did not consider postoperative extraocular muscle expansion (10). Proptosis reduction is due to the combined effect of orbital space expansion from orbital wall decompression and reduction of orbital content from fat decompression. Thus, IREOV and fat decompression are considered the main factors for proptosis reduction. This study compared the correction of proptosis following MWD and LWD and found no statistically significant difference between the two groups. Further analysis demonstrated that IREOV in MWD was significantly greater than that in LWD, while LWD removed more intraorbital fat, achieving a similar effect in proptosis reduction. This also confirmed the importance of orbital fat decompression in reducing proptosis (24, 29). Furthermore, the extent of surgical reduction depends on the levels of ocular proptosis measured preoperatively. Despite prior research focusing on the degree of proptosis reduction achieved by different surgical methods, it is important to note that not all surgeries aim to maximize proptosis reduction. Surgeons must regulate the extent of proptosis reduction during surgery to prevent both excessive and inadequate correction, as maintaining an aesthetically pleasing appearance is essential. The degree of proptosis reduction often depends on the surgeon’s control over the amount of fat extracted during the surgery. It was found that adjusting the amount of fat removed during surgery allows both MWD and LWD surgical methods to achieve similar results in controlling proptosis reduction.

Based on our analysis, we have summarized surgical method selection for TAO patients with moderate proptosis who meet the inclusion criteria of this study. Although MWD achieves a significantly higher IREOV than LWD, resulting in greater orbital volume expansion, LWD can provide similar proptosis reduction by controlling the amount of orbital fat removed. However, for patients with predominant fat hyperplasia, particular caution is needed when performing MWD due to the risk of postoperative extraocular muscle expansion, which may occupy additional orbital volume and affect IREOV and surgical outcomes. Therefore, for TAO patients with fat hyperplasia, LWD should be prioritized to minimize the risk of postoperative muscle expansion.

The limitations of this study include its retrospective nature. Only two single-wall decompression techniques, MWD and LWD, were examined and compared. Future research should expand the application of IREOV to evaluate the outcomes of one-, two-, three-, or four-wall orbital decompression, with or without orbital fat removal, to provide a comprehensive assessment across all decompression techniques. A future prospective, randomized, controlled study with a larger sample size is needed to validate and strengthen our findings, supporting ophthalmologists in selecting optimal surgical options and improving the accuracy of outcome predictions.

## Conclusion

Accurate calculation of IREOV through artificial intelligence technology is essential for assessing the impact of postoperative extraocular muscle expansion on the effectiveness of orbital decompression surgery. Our findings indicate that, for TAO patients with predominant fat hyperplasia, prioritizing LWD can effectively reduce the risk of postoperative muscle expansion. This artificial intelligence technology-based evaluation offers valuable guidance for selecting surgical techniques and optimizing patient outcomes.

## Data Availability

The original contributions presented in the study are included in the article/supplementary material. Further inquiries can be directed to the corresponding authors.
